# Supplementary material for: A Novel Beta-Glucosidase Gene for Plant Type Was Identified by Genome-Wide Association Study and Gene Co-Expression Analysis in Widespread Bermudagrass
Source: Int J Mol Sci. 2022 Sep 28;23(19):11432. doi: 10.3390/ijms231911432 (PMC9570203; doi:10.3390/ijms231911432)
Supplement: Supplementary file 1 [file ijms-23-11432-s001.zip › ijms-1906163-Supplementary materials.pdf]

Supplementary materials

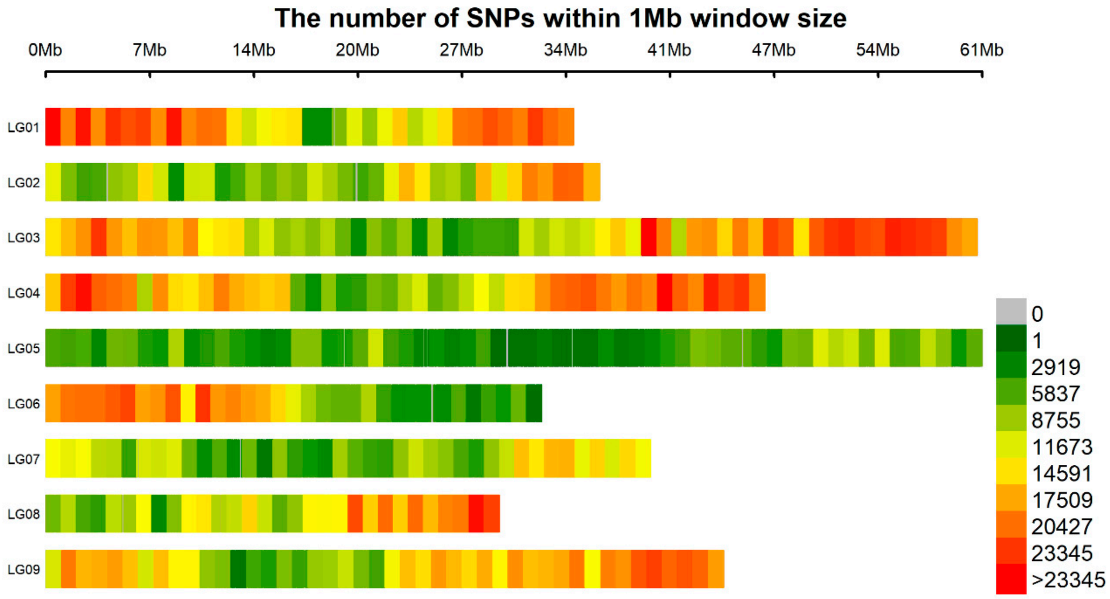

Figure S1. The number of SNPs in chromosomes within 1Mb window size.

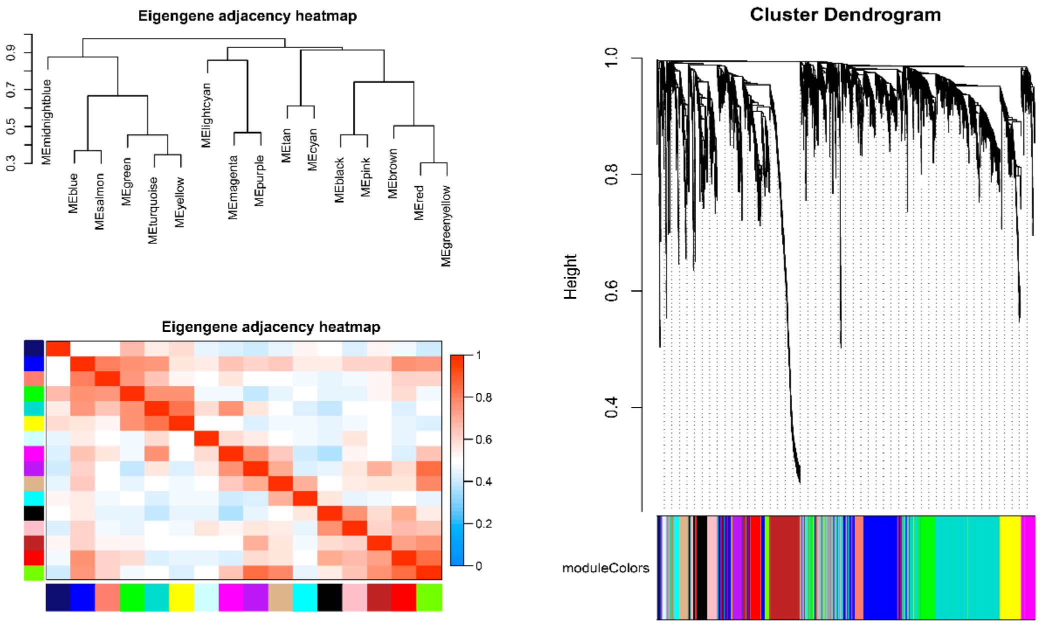

Figure S2. The topological matrix and hierarchical clustering tree of WGCNA.

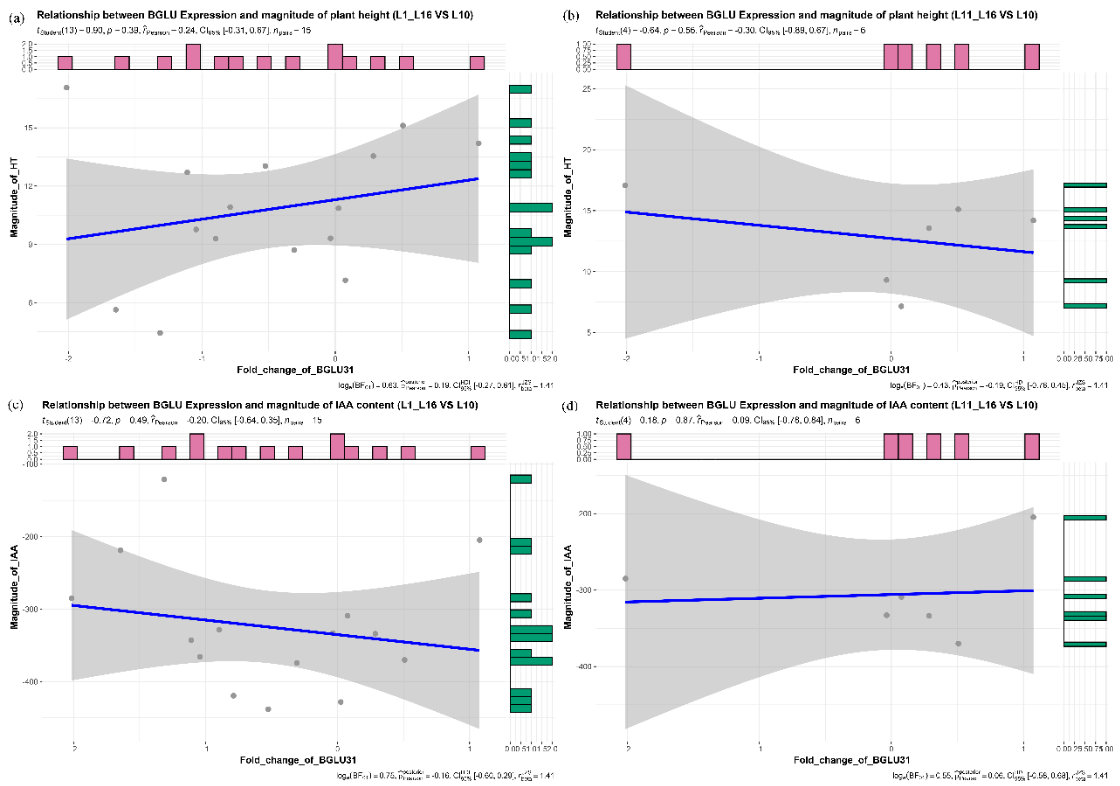

Figure S3. Correlation coefficient analysis between plant traits and BGLU31 expression. (a) Relationship between BGLU Expression and magnitude of plant height (L1_L16 VS L10). (b) Relationship between BGLU Expression and magnitude of plant height (L11_L16 VS L10). (c) Relationship between BGLU Expression and magnitude of IAA content (L1_L16 VS L10). (d) Relationship between BGLU Expression and magnitude of IAA content (L11_L16 VS L10).

```
CLUSTAL O(1.2.4) multiple sequence alignment

CdBGLU*|this                  MAPL-----CFSVLRR----LPFLSLAVVVVVVSVVSREASALTRLDFPAGFVFGAGSSA 51
BAH00605.1|OsBGL31            MTPARVVFICCVVLL---AAA----------AAAASSSTAAGITRADFPPEFIFGAGSSA 47
Q0J0G1                        ---M-----ATGELAL----VSSLFIVVVFLLLGAVAREASALTRHDFPEGFVFGAGSSA 48
Q0J0G2                        ---M-----AAGARAL----VPSPFIVVVFLLLAAAARDASALTRHDFPEGFVFGAGTSA 48
XP_015612589.1|OsBLGU32-like  ---M-----AAGARAL----VPSPFIVVVFLLLAAAARDASALTRHDFPEGFVFGAGTSA 48
NP_001183742.1|ZmBGLU31-like  MMGRKAPLGCATTLLLLLAAAVAPAELSVLGLGGGAAAASGAVTRADFPAGFVFGVGSSA 60
PWZ15718.1|ZmBGLU31           MMGRK-ALGCAPLL-LLLAAAVAPAELSV-----GAAAASGAVTRADFPAGFVFGVGSSA 53
P14228                        -------------MKL----LSNSLMFLPLLALALTAVSSLKYSRNDFPPGFVFGSGTSA 43
                                                                   :  :    :* ***  *:** *:**

CdBGLU*|this                  FQVEGAAAEDGRKPSVWDTFIHAGYSYDGSTADVSADEYHHYKEDVKLMHEMGLDAYRFS 111
BAH00605.1|OsBGL31            YQVEGAFAEDGRKPSIWDTFSHSGYSVDGATGDVTADQYHKYKEDVKLLQDMGVDAYRMS 107
Q0J0G1                        FQVEGAAAEDGRKPSIWDTFINQGYMPDGSNADVSADQYHHYKEDVKLMYDMGLDAYRFS 108
Q0J0G2                        FQVEGAAAEDGRKPSIWDTFTHQGYSPGGAIADVSADQYHHYKEDVKLMYDMGLDAYRFS 108
XP_015612589.1|OsBLGU32-like  FQVEGAAAEDGRKPSIWDTFTHQGYSPGGAIADVSADQYHHYKEDVKLMYDMGLDAYRFS 108
NP_001183742.1|ZmBGLU31-like  YQVEGAVAEDGRKPSIWDTFTHEGYSLDNATGDVTADQYHKYKDDVKLLHEMGVDAYRMS 120
PWZ15718.1|ZmBGLU31           YQVEGAVADDGRKPSIWDTFTHEGYSLDNATGDVTADQYHKYKDDVKLLHEMGVDAYRMS 113
P14228                        YQVEGAADEDGRTPSIWDVFAHAGHS-GVAAGNVACDQYHKYKEDVKLMADMGLEAYRFS 102
                              :*****  :***.**:**.* : *:  . : .:*:.*:**:**:****: :**::***:*

CdBGLU*|this                  IAWPRLIPDGRGEINPKGLEYYNNLIDELILHGIEPHATIYHFDLPQVLQDEYSGLLSPR 171
BAH00605.1|OsBGL31            ISWSRLIPDGRGAVNPKGLEYYNNLIDELLSHGIQPHVTIYHFDFPQALQDEYNGILSPR 167
Q0J0G1                        IAWPRLIPDGRGEINPKGLEYYNNLIDELIMHGIQPHVTIYHFDLPQALQDEYGGILSPR 168
Q0J0G2                        IAWPRLIPDGRGEINPKGLEYYNNLIDELIMHGIQPHVTIYHFDLPQALQDEYGGILSPR 168
XP_015612589.1|OsBLGU32-like  IAWPRLIPDGRGEINPKGLEYYNNLIDELIMHGIQPHVTIYHFDLPQALQDEYGGILSPR 168
NP_001183742.1|ZmBGLU31-like  IAWPRLIPDGRGAVNPKGLEYYNNLIDELLSYGIQPHVTIYHFDFPQALQDEYSGLISPR 180
PWZ15718.1|ZmBGLU31           IAWPRLIP------------------------GIQPHVTIYHFDFPQALQDEYSGLISPR 149
P14228                        ISWSRLLPSGRGPINPKGLQYYNNLIDELITHGIQPHVTLHHFDLPQALEDEYGGWLSQE 162
                              *:* **:*                        **:**.*::***:**.*:***.* :* .

CdBGLU*|this                  FIEDYVAFANVCFHNFGDRVKHWVTVNEPNIETIGGYDVGAQPPRRCSYPFGE-NCTAGN 230
BAH00605.1|OsBGL31            FVEDFTAYADVCFKNFGDRVKHWSTVNEPNIEPIGGYDQGILPPRRCSFPFGVLSCDNGN 227
Q0J0G1                        FIEDYSAYAEVCFKNFGDRVKHWATFNQPNIEPIGGFDAGDRPPRRCSYPFGT-NCTGGD 227
Q0J0G2                        FIEDYTAYAEVCFKNFGDRVKHWVTVNEPNIEPIGGYDAGVQPPRRCSYPFGT-NCTGGD 227
XP_015612589.1|OsBLGU32-like  FIEDYTAYAEVCFKNFGDRVKHWVTVNEPNIEPIGGYDAGVQPPRRCSYPFGT-NCTGGD 227
NP_001183742.1|ZmBGLU31-like  FIEDFTAYADVCFSNFGDRVKYWSTVNEPNVETIGGYDQGILPPRRCSFPFGF-GCEEGN 239
PWZ15718.1|ZmBGLU31           FIEDFTAYADVCFSNFGDRVKYWSTVNEPNVETIGGYDQGILPPRRCSFPFGF-GCEEGN 208
P14228                        IVRDFTAYADTCFKEFGDRVSHWTTINEVNVFALGGYDQGITPPARCSPPFGL-NCTKGN 221
                              ::.*: *:*:.** :*****.:* *.*: *:  :**:* *  ** *** ***  .*  *:

CdBGLU*|this                  SSTEPYIAAHHLLLAHAAAVSLYRDKYKG------------------------------- 259
BAH00605.1|OsBGL31            STTEPYIVAHHLLLAHSSAVSLYREKYQAT----------------------QGGQIGL 264
Q0J0G1                        SSTEPYIVAHHLLLAHASAVSIYRQKYQAI----------------------QGGQIGI 264
Q0J0G2                        SSTEPYIVAHHLLLAHASAVSIYRQKYQAI----------------------QGGQIGI 264
XP_015612589.1|OsBLGU32-like  SSTEPYIVAHHLLLAHASAVSIYRQKYQAI----------------------QGGQIGI 264
NP_001183742.1|ZmBGLU31-like  STTEPYVAAHHLLLAHASAVSLYRDRYQAA----------------------QGGRIGL 276
PWZ15718.1|ZmBGLU31           STTEPYVAAHHLLLAHASAVSLYRDRYQAE----------------------QGGRIGL 245
P14228                        SSIEPYIAVHNMLLAHASATILYKQQYKVLLSASLPSSICIALCYVLFITQYKQHGSVGI 281
                              *: ***:..*::****::*.  :*:::*:

CdBGLU*|this                  ------------------------------------------------------------
BAH00605.1|OsBGL31            TLLGWWYEPGTQDPEDVAAAARMNDFHIGWYMHPLVYGDYPPVMRKNVGSRLPSFTAEES 324
Q0J0G1                        TLMVRWHEPYTDKTADAAAAIRMNEFHIGWFLHPLVHGDYPPVMRSRVGVRLPSITASDS 324
Q0J0G2                        TLLGWWYEPYTDAVADAAAAIRMNEFHIGWFMNPLVHGDYPPVMRSRVGARLPSITASDS 324
XP_015612589.1|OsBLGU32-like  TLLGWWYEPYTDAVADAAAAIRMNEFHIGWFMNPLVHGDYPPVMRSRVGARLPSITASDS 324
NP_001183742.1|ZmBGLU31-like  TLLGWWYEPGTQTPDDVAAAARMNDFHIGWFMHPMVFGDYPPVMRRNVGSRLPTFTDEEA 336
PWZ15718.1|ZmBGLU31           TLLGWWYEPGTQTPDDVAAAARMNDFHIGWFMHPMVFGDYPPVMRRNVGSRLPTFTDEEA 305
P14228                        SVYTYGAVPLTNSVKDKQATARVNDFYIGWILHPLVFGDYPETMKTNVGSRLPAFTEEES 341

CdBGLU*|this                  ------------------------------------------------------------
BAH00605.1|OsBGL31            KRVLESYDFVGFNHYVAIFVRADLSKLDQSLRDYMGDAAVKYDLPFLKSNNEFPLG-LTS 383
Q0J0G1                        EKIRGSFDFIGINHYYVIFVQSIDAN-EQKLRDYYIDAGVQGE-------------DDT 369
Q0J0G2                        EKIRGSFDFIGINHYFVIFVQSSDANHDQKLRDYYVDAGVQENG----------GGGFDK 374
XP_015612589.1|OsBLGU32-like  EKIRGSFDFIGINHYFVIFVQSSDANHDQKLRDYYVDAGVQENG----------GGGFDK 374
NP_001183742.1|ZmBGLU31-like  ARVRGSFDFVGFNHYIVVYVKADLGRLDDQVRDYMGDAAVKYDMPFLKSRNQFPFGALTS 396
PWZ15718.1|ZmBGLU31           ARVRGSFDFVGFNHYIVVYVKADLGRLDDQVRDYMGDAAVKYDMPFLKSRNQFPFGGLTS 365
P14228                        EQVKGAFDFVGVINYMALYVKDNSSSLKPNLQDFNTDIAVEMTLV---------GNTSIE 392

CdBGLU*|this                  -EIESPPWALGKLLDHLKLKYENPLVFIHENGAGEAPDPPGAIVYDDEFRSEMLQDYLEV 318
BAH00605.1|OsBGL31            DFMTSTPWALKKMLNHLQEKYKNPIVMIHENGAAGQPDPSGGNTYDDDFRSQYLQDYIEA 443
Q0J0G1                        ENIQCHSWSLGKVLNHLKLEYGNPPVMIHENGYSDSPDIFGKINYNDDFRSAFLQGYLEA 429
Q0J0G2                        EHYQLHPWALGKMLHHLKLKYGNPPVMIHENGDADSPETPGKIDYDDDFRSDFLQSYLEV 434
XP_015612589.1|OsBLGU32-like  EHYQLHPWALGKMLHHLKLKYGNPPVMIHENGDADSPETPGKIDYDDDFRSDFLQSYLEV 434
NP_001183742.1|ZmBGLU31-like  DFMTSTPWALKKMLRHLRVTYKNPAVMIHENGAAGQPDPSGVNSYDDEFRSQFLQDYIEA 456
PWZ15718.1|ZmBGLU31           DFMTSTPWALKKMLRHLRVTYKNPAVMIHENGAAGQPDPSGVNSYDDEFR---------- 415
P14228                        NEYANTPWSLQQILLYVKETYGNPPVYILENGQMTPH----SSSLVDTTRVKYLSSYIKA 448
                                     *:* ::* :::  * ** * * ***             *  *

CdBGLU*|this                  LHMSIRNGSNARGYFVWSFLDVFEFLFAYRVRFGLCGVDMNAAARTRYVRSSARWYAGFL 378
BAH00605.1|OsBGL31            TLQSTRNGSNVQGYFVWSFLDVFEYLFGYRLRFGLYGVDFASPERTRYQRHSARWYAGFL 503
Q0J0G1                        LYLSVRNGSNTRGYFVWSMFDMFEFLYGYRLRFGLCGVDFTAAARTRYLKNSARWYSGFL 489
Q0J0G2                        LHLSIRNGSNTRGYFVWSLLDGFEFLSGYGNRFGLCCVDFTAPARTRYVRSSARWYSDFL 494
XP_015612589.1|OsBLGU32-like  LHLSIRNGSNTRGYFVWSLLDGFEFLSGYGNRFGLCCVDFTAPARTRYVRSSARWYSDFL 494
NP_001183742.1|ZmBGLU31-like  TLQSIRNGSNVQGYFVWSFLDVFEYLFGYRLRFGVYGVEFNSTARTRYQRHSAKWYASFL 516
PWZ15718.1|ZmBGLU31           ------NGSNVQGYFVWSFLDVFEYLFGYRLRFGVYGVEFNSTARTRYQRHSAKWYASFL 469
P14228                        VLHSLRKGSDVKGYFQWSLMDVFELFGGYERSFGLLYVDFKDPSLKRSPKLSAHWYSSFL 508
                                    :**:.:*** **::* ** : .*   **:  *::     .*  : **:**:.**

CdBGLU*|this                  RCGELRPPAFRPDNRSYVADE 399
BAH00605.1|OsBGL31            RGGELRPAAAALAGGGAYSQ- 523
Q0J0G1                        RGGELRPEKSYATL------- 503
Q0J0G2                        NGGELRPVKPFVAL------- 508
XP_015612589.1|OsBLGU32-like  NGGELRPVKPFVAL------- 508
NP_001183742.1|ZmBGLU31-like  RGGELRPVALP---DGAYSQ- 533
PWZ15718.1|ZmBGLU31           RGGELRPVALP---DGAYSQ- 486
P14228                        KGTLHHPSYASS--------- 520
                              .    :*
```

Figure S4. Alignment analysis of BGLU gene from different plants.

Table S1. Geographical locations, plant height and IAA content of diverse bermudagrass

**Region**	**Latitude**	**Sample**	**HT**	**IAA**
**Subtropical zone**	22°35′40″	L1	20.43	266.92438
	22°51′48″	L2	17.49	244.04445
	24°10′31″	L3	20.76	171.6514
	25°05′29″	L4	18.64	190.29881
	26°03′49″	L5	18.58	181.54994
	27°00′59″	L6	17.01	281.34448
	28°09′14″	L7	16.42	235.61128
	29°28′32″	L8	13.36	390.85805
	30°25′48″	L9	12.16	488.7188
	31°18′59″	L10	7.72	609.71927
**Mesotherm zone**	32°08′38″	L11	14.87	300.4336
	33°09′47″	L12	17.03	276.79077
	34°00′30″	L13	21.27	275.84688
	34°54′04″	L14	22.84	239.66685
	35°29′26″	L15	21.92	405.10552
	36°18′40″	L16	24.79	324.74452
**Coefficient**	Total region	L1-L16	0.156	0.410
	Subtropics	L1-L10	-0.890	0.792
	Mesotherm	L11-L16	0.957	0.387

Table S2. Detailed data of GO analysis result of DEGs.

GO ID	Description	DEGs	All Gene	pvalue	p.adjust
GO:0044710	single-organism metabolic process	390	2070	1.07E-07	0.0001002
GO:0055114	oxidation-reduction process	221	1167	0.0001239	0.0165487
GO:1901615	organic hydroxy compound metabolic process	8	12	8.04E-05	0.0150624
GO:0006082	organic acid metabolic process	70	307	0.0002416	0.0208166
GO:0043436	oxoacid metabolic process	70	306	0.000218	0.0208166
GO:0019752	carboxylic acid metabolic process	70	300	0.0001155	0.0165487
GO:0044281	small molecule metabolic process	106	452	1.55E-06	0.0007257
GO:0006520	cellular amino acid metabolic process	43	153	2.97E-05	0.0092672
GO:0043038	amino acid activation	21	66	0.000546	0.0283608
GO:0006334	nucleosome assembly	10	20	0.0002822	0.0208166
GO:0006323	DNA packaging	10	21	0.0004655	0.0272037
GO:0071824	protein-DNA complex subunit organization	11	23	0.0002299	0.0208166
GO:0031497	chromatin assembly	10	20	0.0002822	0.0208166
GO:0006334	nucleosome assembly	10	20	0.0002822	0.0208166
GO:0034728	nucleosome organization	11	21	8.05E-05	0.0150624
GO:0006066	alcohol metabolic process	7	11	0.0003562	0.023791
GO:1901615	organic hydroxy compound metabolic process	8	12	8.04E-05	0.0150624
GO:0003824	catalytic activity	1005	6224	1.70E-06	0.0009385
GO:0048037	cofactor binding	78	339	2.52E-05	0.0057297
GO:0050662	coenzyme binding	61	250	3.11E-05	0.0057297

Table S3. Phenotypic characterizations of *bglu11* mutant and wild type of Arabidopsis

	WT	M1	M2
Height of rosette	1.99±0.09	1.81±0.05	1.63±0.04
Diameter of rosette	5.26±0.03	4.73±0.05	3.12±0.07
Plant Height	16.38±0.55	7.57±0.24	9.75±0.12
